# Alterations in DNA Methylation May Be the Key to Early Detection and Treatment of Schistosomal Bladder Cancer

**DOI:** 10.1371/journal.pntd.0003696

**Published:** 2015-06-04

**Authors:** Simon L. Conti, Jared Honeycutt, Justin I. Odegaard, Mark L. Gonzalgo, Michael H. Hsieh

**Affiliations:** 1 Department of Urology, Stanford University, Stanford, California, United States of America; 2 Stanford Immunology, Stanford University, Stanford, California, United States of America; 3 Cardiovascular Research Institute, University of California San Francisco, San Francisco, California, United States of America; 4 Department of Urology, University of Miami Miller School of Medicine, Miami, Florida, United States of America; 5 Biomedical Research Institute, Rockville, Maryland, United States of America; 6 Division of Urology, Children’s National Health System, Washington, D.C., United States of America; 7 Department of Urology, The George Washington University, Washington, D.C., United States of America; Centers for Disease Control and Prevention, UNITED STATES

Bladder cancers arise from transformed urothelial cells that line the bladder. These cancers are urothelial or squamous cell carcinomas or, more rarely, additional histologic variants such as adenocarcinoma. The most important bladder cancer risk factors worldwide are arguably smoking and urogenital schistosomiasis. The parasitic *Schistosoma haematobium* worm causes urogenital schistosomiasis in approximately 112 million people, primarily in sub-Saharan Africa and the Middle East [1]. During infection, *S*. *haematobium* worms lay highly inflammatory eggs in the bladder wall. This inflammation is thought to promote carcinogenesis through unclear mechanisms. People with chronic urogenital schistosomiasis exhibit increased risk and earlier onset of bladder cancer (up to two decades earlier), with a predominance of squamous cell carcinoma [2]. Consequently, *S*. *haematobium* has been categorized as a Group I carcinogen (“carcinogenic to humans”) by the International Agency on Research on Cancer of the World Health Organization [3].

Although *S*. *haematobium* is an accepted bladder carcinogen, many patients with schistosomal bladder cancer present at advanced stages [4]. We posit that this is due to a combination of poor medical infrastructure in endemic areas and a lack of diagnostic and prognostic tools for schistosomal bladder cancer and its precursor epigenetic events.

One promising strategy for improving the diagnosis and prognostication of schistosomal bladder cancer is analysis of urothelial DNA methylation. This epigenetic modification, in which cytosine bases are converted to methylcytosines, is becoming increasingly appreciated as a source of carcinogenesis in multiple cancers, including leukemias, lymphomas, and bladder, colon, and esophageal carcinomas [5,6]. Not surprisingly, DNA methylation also has been implicated in preneoplastic changes in many of these same tissues, e.g., in the colon and esophagus (reviewed in [7]). Furthermore, there is evidence that DNA methylation changes may also be elicited by uropathogenic *Escherichia coli* infections, a known risk factor for bladder cancer (as in the well-established case of *Helicobacter pylori* in gastric carcinogenesis) [8]. One major pathway by which DNA methylation likely contributes to carcinogenesis is reversible, promoter methylation-induced silencing of expression of tumor suppressor genes [7]. Over time, promoter methylation-induced silencing of expression of tumor suppressor genes may lead to accumulation of additional procarcinogenic DNA methylation events and outright mutations.

Indeed, DNA hypermethylation of numerous genes, including the tumor suppressor genes *RASSF1A* and *TIMP3*, has been identified in the urine of patients with urogenital schistosomiasis[9]. Other investigators have identified DNA methylation of *RARbeta2* and *APC* as potential urine biomarkers of bladder cancer, with schistosomiasis-associated cases having higher rates of methylation of these genes [10]. Yet another group reported differential methylation of *CDH1*, *DAPK1*, *CDKN2A*, *MGMT*, *ICDKN2B*, *FHIT*, *APC*, *RASSF1*, *GSTP1*, *RARB*, and *TP73* in bladder cancer specimens, with schistosomiasis-associated specimens featuring more differentially methylated genes than those not associated with this infection [11]. Interestingly, Saad et al. reported that levels of N7-methylguanine, a form of methylated guanine, were more frequently elevated in cancerous versus normal bladder tissues from patients with bladder cancer, regardless of whether it was associated with schistosomiasis or not [12]. However, this marker was not further increased in the subset of patients with schistosomal bladder cancer (relative to non-schistosomiasis-associated cancers), suggesting that DNA methylation is not unique to schistosomal bladder cancer. Regardless, hypermethylation of genes (especially tumor suppressor genes) may be a key cause of reversible preneoplastic lesions of the bladder urothelium exposed to *S*. *haematobium* infection.

Despite the importance of *S*. *haematobium* as a bladder carcinogen, studies of the associated basic cancer biology (including the role of DNA methylation) have been limited because of a historical lack of tractable animal models for urogenital schistosomiasis. To address the need for tractable tools to study schistosomal cancer biology, we developed the first experimentally tractable mouse model of urogenital schistosomiasis [13]. In this model, microinjection of *S*. *haematobium* parasite eggs into the bladder walls of mice leads to rapid urothelial hyperplasia [13] and squamous metaplasia. Indeed, urothelial hyperplasia persists for at least 3 months after egg exposure [13]. Thus, our approach recapitulates key preneoplastic changes in the bladder associated with urogenital schistosomiasis.

To identify what bladder urothelial DNA methylation events occur in the preneoplastic period in our mouse model of urogenital schistosomiasis, we microinjected *S*. *haematobium* eggs into the bladder walls of mice and 2 weeks later microdissected the urothelium of each bladder from the remaining bladder tissue. The granuloma and the detrusor tissue were discarded (S1 Fig). Granuloma formation was noted in all *S*. *haematobium*-injected bladders. We then used reduced representation bisulfite sequencing (RRBS) [14,15] to profile DNA methylation across the urothelial genome. RRBS focuses on methylation of cytosines within CpG dinucleotide “islands,” which are genomic regions enriched for potential DNA methylation sites. In brief, DNA was extracted from each specimen and the restriction enzyme Msp1 used to fragment DNA at CpG islands. After bisulfite treatment samples were fragmented to a length of 175–225 bp, they were then amplified by PCR. Multiplexed sequencing was performed using the Illumina Hi-Seq platform. The output was aligned to the Mouse Genome Reference Consortium *Mus musculus* genome (GRCm38/mm10) using Bismark software. Methylation analysis was performed with methylKit and the Integrative Genomics Viewer (IGV).

Using thresholds of >10x coverage, >25% difference in methylation, and *p* < 0.05, we found that short-term exposure of the mammalian bladder to *S*. *haematobium* infection led to massive changes in DNA methylation of the urothelium. *S*. *haematobium* egg-injected mice featured major alterations in their methylome (versus control mice) 2 weeks post-egg exposure (Fig 1). 13,333 cytosines were hypermethylated, and 6,244 were hypomethylated. These data were processed using a promoter identification algorithm and subsequently fed into Database for Annotation, Visualization and Integrated Discovery (DAVID) pathways analysis. Of these differentially methylated cytosines, 1,019 were found to be within 1,000 base pairs of a transcription start site for a known gene (i.e., putative promoter regions). Six of these genes are part of the Wnt canonical pathway (Fig 2), which is related to cell proliferation. A CpG in the promoter of the Wnt inhibitory factor-1 (*Wif1*) gene, a gene silenced by hypermethylation in bladder tumors and other cancers [16,17], was methylated 54% of the time in egg-injected mice, 34% in nitrosamine-fed mice, and 7% in control mice. Thus, even short-term exposure of the mammalian bladder to *S*. *haematobium* eggs results in profound alterations in DNA methylation, including in the promoters of known tumor suppressor genes such as *Wif1*. Indeed, through qPCR we discovered that WIF-1 expression was decreased in mouse urothelia after subepithelial bladder injections with eggs versus control vehicle (0.58-fold expression in six egg-injected mice compared to two control-injected mice, 95% CI 0.475–0.713). Primary sequencing data can be found at http://www.ncbi.nlm.nih.gov/bioproject/PRJNA278470.

**Fig 1:**
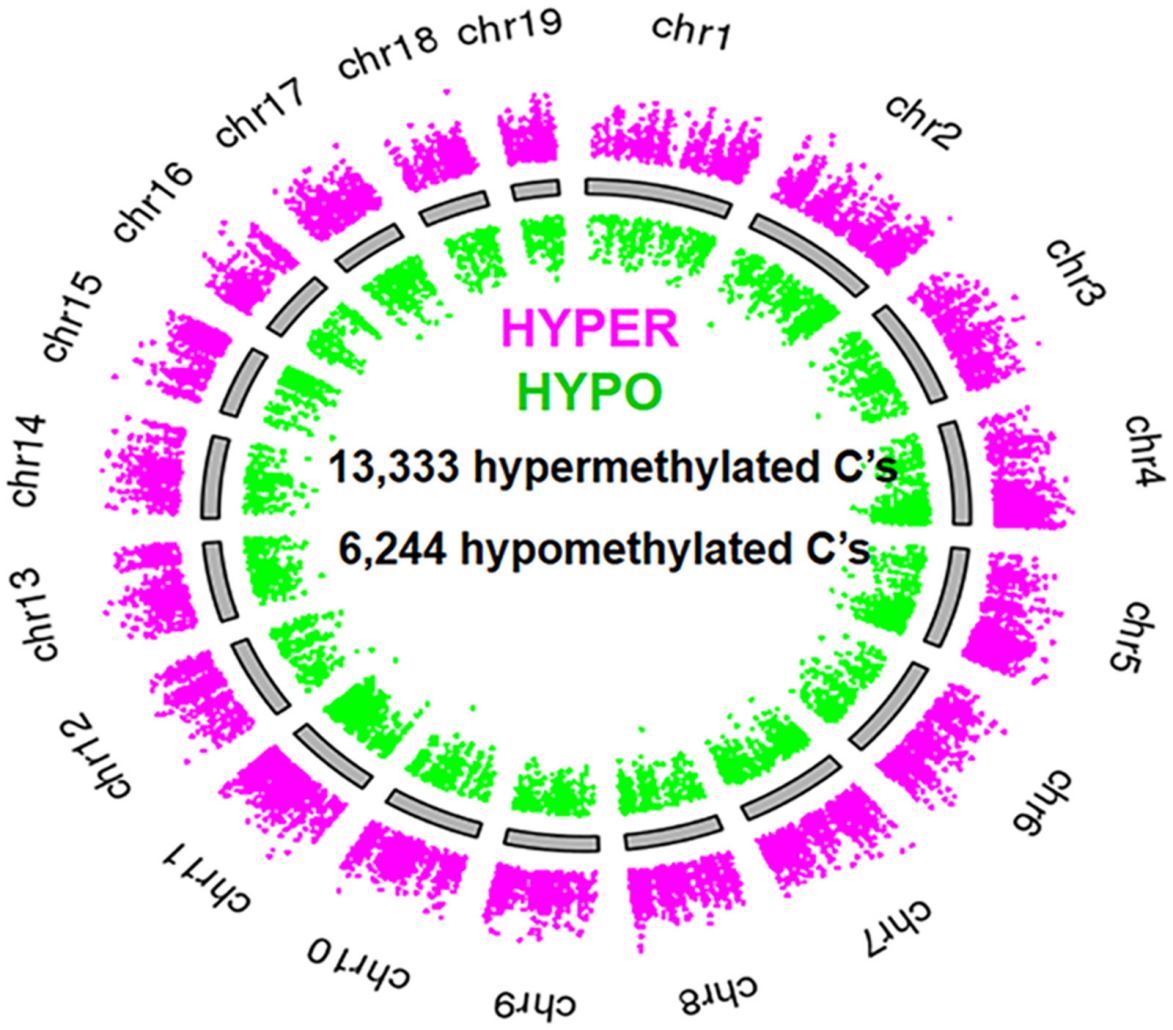
Exposure of the bladder to *S*. *haematobium* eggs induces massive shifts in the urothelial methylome. Genome map demonstrating that multiple loci are hypermethylated (magenta dots) and hypomethylated (green dots) in the mouse urothelium exposed to *S*. *haematobium* eggs versus vehicle controls. Representative map from one of two experimental replicates shown.

**Fig 2:**
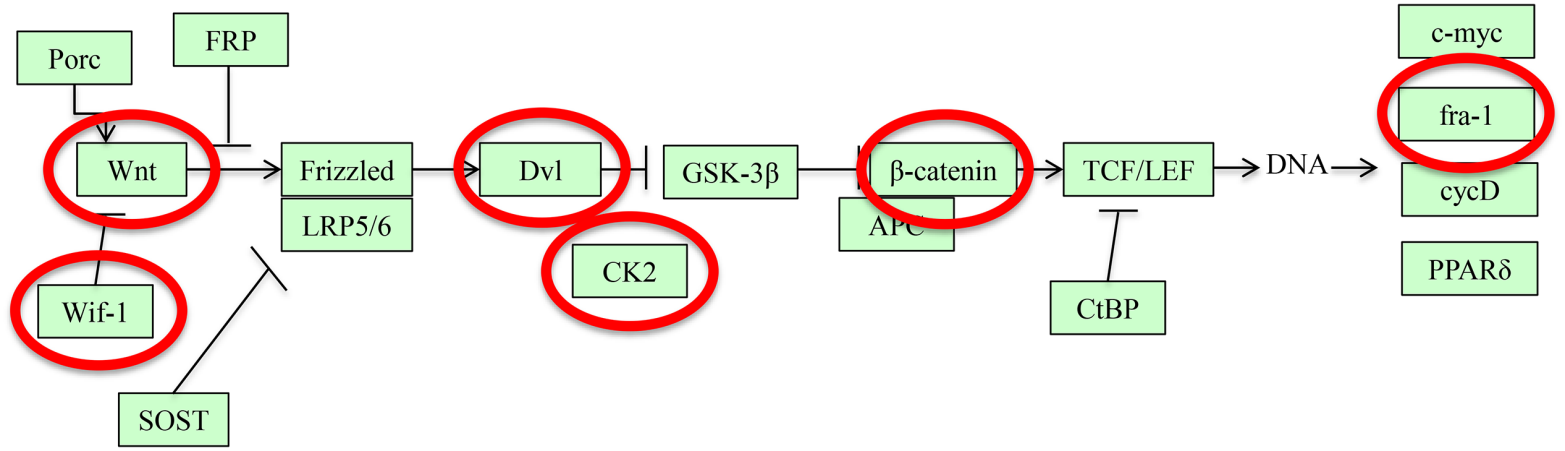
*S*. *haematobium* eggs induce differential methylation of multiple members of the Wnt signaling pathway, including the bladder cancer-associated tumor suppressor gene *Wif1*. Differentially methylated gene members of the Wnt signaling pathway are circled in red. Note that *Wif1*, a tumor suppressor gene implicated in bladder carcinogenesis, is differentially methylated and sits far upstream along the Wnt pathway. Figure generated using DAVID (http://david.abcc.ncifcrf.gov/).

Given that *S*. *haematobium* eggs induced differential methylation of many urothelial genes, we sought to reverse this process pharmacologically. We gave the DNA methylation inhibitor 5-fluoro-2ʹ-deoxycytidine (FdCyd, 12.5 mg/kg) and tetrahydrouridine (THU, an inhibitor of FdCyd metabolism, 25 mg/kg) on an every other day basis (14 days total) intraperitoneally to *S*. *haematobium* egg-injected mice. This drug combination suppressed *S*. *haematobium* egg-induced urothelial hyperplasia, a key preneoplastic change in the bladder (Fig 3). Specifically, mice treated with FdCyd and THU had a mean urothelial thickness of 67 μm (±19) and those treated with vehicle injections had a mean thickness of 101 μm (±26, *p* = 0.043—unpaired Student’s *t* test).

**Fig 3:**
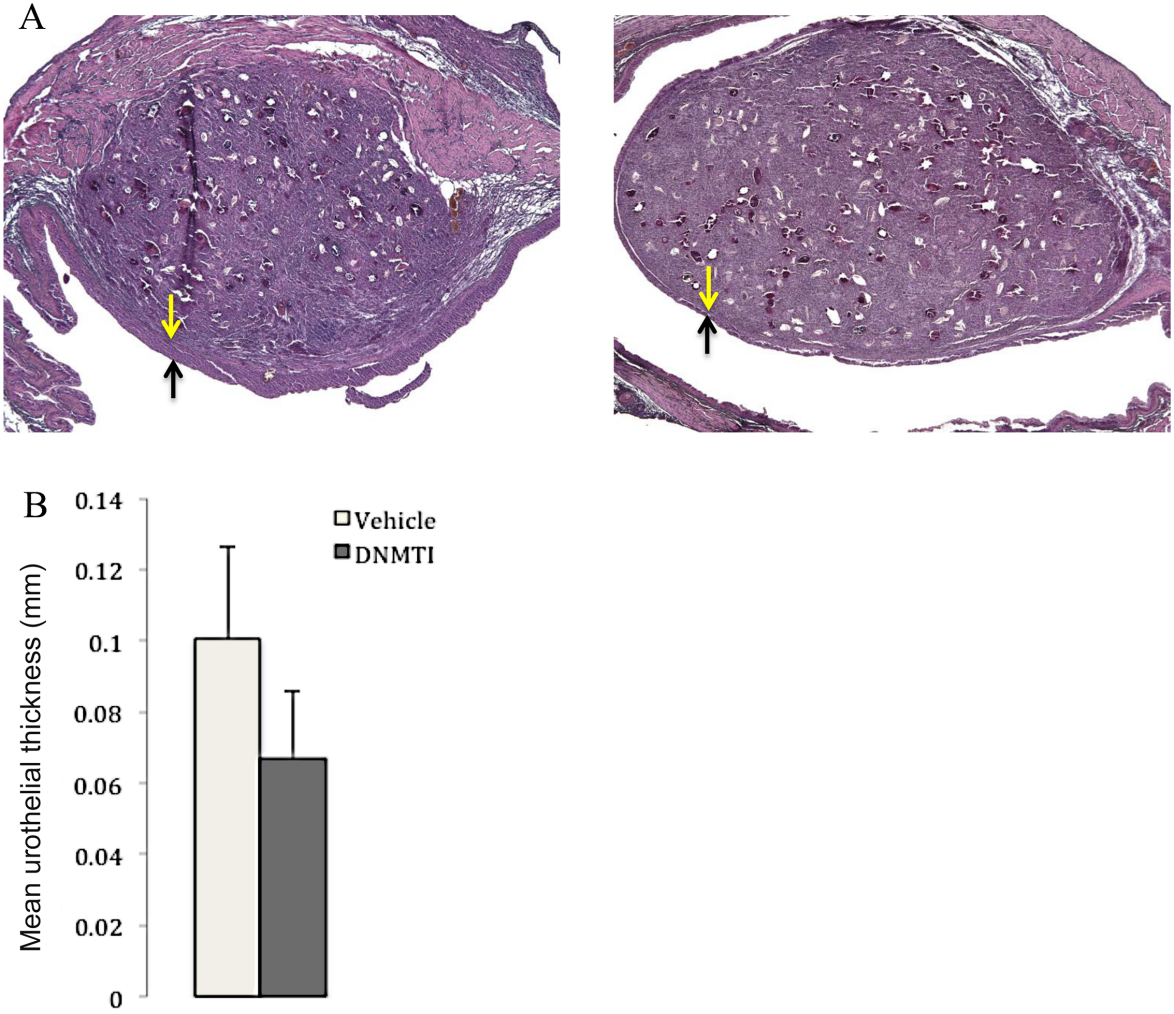
In vivo inhibition of DNA methylation prevents *S*. *haematobium* egg-induced urothelial hyperplasia, a potential preneoplastic lesion of the bladder. (A) Every other day administration of the DNA methylation inhibitor 5-fluoro-2ʹ-deoxycytidine (FdCyd) (12.5 mg/kg) and tetrahydrouridine (THU, 25 mg/kg, an inhibitor of metabolism of FdCyd) for 14 days prevents *S*. *haematobium* egg-induced urothelial hyperplasia (*n* = 4 mice, right panel) compared to vehicle control (*n* = 3 mice, left panel). The yellow and black arrows denote the thickness of the urothelium overlying the egg-induced bladder granuloma. (B) Bar graph depicting urothelial thickness from (A) in graphical format (“DNMTI” and “Vehicle” indicate the DNA methylation inhibitor- and vehicle-treated groups, respectively).

To our knowledge, this is the first in vivo demonstration that pharmacologic inhibition of DNA methylation can prevent preneoplastic changes in the bladder. Thus, manipulating DNA methylation may be a promising approach to slow or prevent bladder carcinogenesis in high-risk patient populations, namely those with extensive exposure to carcinogens such as urogenital schistosomiasis. Mass drug administration campaigns, snail control, better water hygiene, and public education regarding schistosomiasis are likely to have the greatest impact on bladder cancer associated with *S*. *haematobium*. However, a period of chronic bladder inflammation caused by parasite eggs may be enough to predispose individuals to subsequent metaplastic changes, even after successful treatment [18].

Admittedly, our suggestions are highly speculative, and much more detailed work is required to characterize any mechanistic relationships between urogenital schistosomiasis-induced urothelial DNA methylation and bladder oncogenesis. Nonetheless, there are indications that chronic cystitis and squamous cell carcinoma of the bladder, both linked to urogenital schistosomiasis, may have causal associations with bladder urothelial DNA methylation [19,20]. If a relationship between *S*. *haematobium* infection-mediated bladder urothelial DNA methylation and bladder oncogenesis is confirmed, then we propose that it may be possible to identify *S*. *haematobium*-infected or former patients at high risk of bladder carcinogenesis through urine testing for urothelial DNA methylation patterns. Administration of DNA methylation inhibitors such as FdCyd, which are currently in clinical trials for urothelial carcinoma, could then be evaluated for their ability to reduce the risk of these patients developing advanced schistosomal bladder cancer.

## Ethics Statement

All animal work was conducted according to relevant United States and international guidelines. Specifically, all experimental procedures were carried out in accordance with the Administrative Panel on Laboratory Animal Care (APLAC) protocol and the institutional guidelines set by the Veterinary Service Center at Stanford University (Animal Welfare Assurance A3213-01 and US Department of Agriculture [USDA] License 93-4R-00). Stanford APLAC and institutional guidelines are in compliance with the US Public Health Service Policy on Humane Care and Use of Laboratory Animals. The Stanford APLAC approved the animal protocol associated with the work described in this publication.

## Supporting Information

S1 Fig Dissection of the urothelium of the mouse bladder. (A) An *S*. *haematobium* egg-injected bladder fileted open and immobilized with pins. The black oval denotes a subepithelial egg granuloma. (B) Hematoxylin and eosin staining of the muscular detrusor layer of the bladder, which has been dissected away, leaving the (C) isolated urothelium available for downstream analyses.
(TIF)
