# Supplementary figures and images for: Alterations in DNA Methylation May Be the Key to Early Detection and Treatment of Schistosomal Bladder Cancer
Source: PLoS Negl Trop Dis. 2015 Jun 4;9(6):e0003696. doi: 10.1371/journal.pntd.0003696 (PMC4456143; doi:10.1371/journal.pntd.0003696)

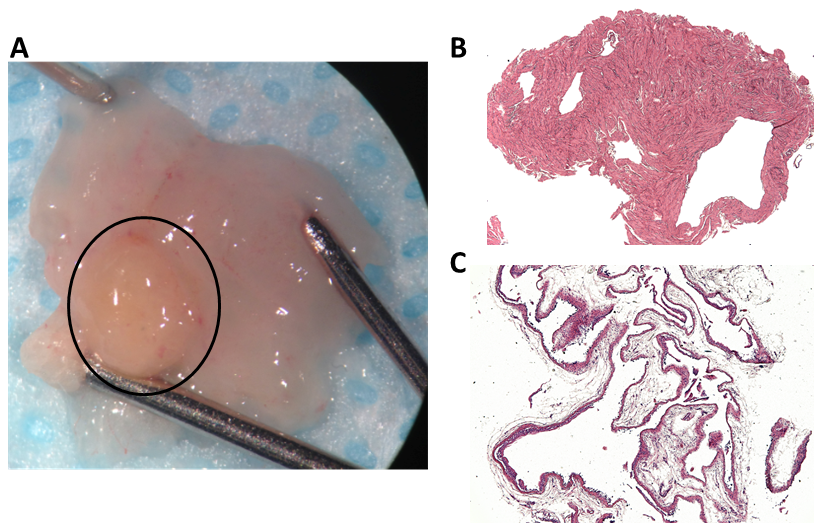

Supplement: S1 Fig — (A) An S. haematobium egg-injected bladder fileted open and immobilized with pins. The black oval denotes a subepithelial egg granuloma. (B) Hematoxylin and eosin staining of the muscular detrusor layer of the bladder, which has been dissected away, leaving the (C) isolated urothelium available for downstream analyses. (TIF) [file pntd.0003696.s001.tif]
